# Open-source Single-particle Analysis for Super-resolution Microscopy with VirusMapper

**DOI:** 10.3791/55471

**Published:** 2017-04-09

**Authors:** Robert D. M. Gray, Jason Mercer, Ricardo Henriques

**Affiliations:** ^1^MRC Laboratory for Molecular Cell Biology, University College London; ^2^Centre for Mathematics and Physics in Life Sciences and Experimental Biology (CoMPLEX), University College London; ^3^Department of Cell and Developmental Biology, University College London

**Keywords:** Molecular Biology, Issue 122, super-resolution microscopy, fluorescence microscopy, image analysis, single-particle analysis, software, viruses, Fiji, vaccinia

## Abstract

Super-resolution fluorescence microscopy is currently revolutionizing cell biology research. Its capacity to break the resolution limit of around 300 nm allows for the routine imaging of nanoscale biological complexes and processes. This increase in resolution also means that methods popular in electron microscopy, such as single-particle analysis, can readily be applied to super-resolution fluorescence microscopy. By combining this analytical approach with super-resolution optical imaging, it becomes possible to take advantage of the molecule-specific labeling capacity of fluorescence microscopy to generate structural maps of molecular elements within a metastable structure. To this end, we have developed a novel algorithm — VirusMapper — packaged as an easy-to-use, high-performance, and high-throughput ImageJ plugin. This article presents an in-depth guide to this software, showcasing its ability to uncover novel structural features in biological molecular complexes. Here, we present how to assemble compatible data and provide a step-by-step protocol on how to use this algorithm to apply single-particle analysis to super-resolution images.

**Figure:**
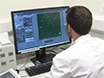


## Introduction

Super-resolution (SR) microscopy has had a major impact on cell biology by providing the ability to image key molecular processes along with the molecular specific labeling crucial to understanding them. SR now enables light microscopy to approach the resolutions (20-150 nm) previously only achievable with electron microscopy (EM) while retaining the major benefits of light microscopy, such as the potential to image live cells^1^,^2^. Further, the structural conservation found at the nanoscale level permits the application of single-particle analysis (SPA) to SR data, a concept used extensively in electron microscopy^3^. Using SPA, many highly conserved copies of a structure can be imaged and averaged together to improve the resolution, precision, or signal-to-noise of the visualized object. When used in combination with SR, SPA has been demonstrated to be a powerful tool for the high-precision mapping of components of the nuclear pore complex^4^,^5^, centrosomes^6^, and viruses, such as HIV^7^ and HSV-1^8^.

However, the routine combined application of SR and SPA has been challenged by a lack of available software. For this reason, we developed VirusMapper, a plugin to the popular image processing software ImageJ/Fiji^9^. This is the first freely available software package for generalized SPA with fluorescence images^10^ designed to provide fast, user-friendly, multi-channel naïve averaging of structures imaged with SR microscopy. Although designed for viruses, it can be applied to any macromolecular complex in which different molecular species can be imaged, identified, and localized.

VirusMapper can be used to produce high-precision molecular models of any known structure, allowing for the calculation of average dimensions and other parameters. The algorithm design makes it particularly useful for separating populations of structures, providing for the determination of distinct orientations or different morphological states. Additionally, multichannel imaging can be used to employ a reference channel in cases where the underlying structure is well-known, thereby allowing for reference-based structure discovery. The instructions for downloading and installing the software are provided on https://bitbucket.org/rhenriqueslab/nanoj-virusmapper. Example data can also be found there, and users are advised to practice using the software on the example data before attempting to apply it to their own.

Here, the steps for using this plugin to produce SPA models from raw data are described. The software takes raw images containing single- or multi-labeled structures as input. It returns, subject to a number of parameters that are adjusted as the software is run, SPA models showing the average distributions of the labeled components within the imaged structures.

The goal of this protocol is to produce precise SPA models giving the average localizations of components within imaged structures according to the pipeline outlined in **Figure 1**. As shown in **Figure 1**, the software workflow is usefully divided into three stages. The first stage is to segment large images, resulting in stacks of particles for each channel. These particles are the units that will be averaged to create models and to produce seeds for model generation. The second stage is to generate seed images, which are used to register the entire set of particles in the final stage. This is done by choosing a reference channel and manually selecting particles in this channel that will contribute to the seeds. Seeds are chosen in this reference channel but can be generated for all channels. Particles are initially realigned by fitting a 2D Gaussian in this channel. All particles that have been selected and realigned are then averaged to produce a seed. For each common structure seen in the data that is to be modeled, particles should be selected as seeds that clearly and accurately represent that structure. The interface at this stage is also useful for scanning the data for such structures.

The final stage is to generate models using template matching. This is achieved through the registration of the particles originally extracted to the seed images generated in the previous section by cross-correlation. A subset of registered particles is averaged together, and the process is further iterated to reduce model mean squared error, if desired. This subset is determined by setting a minimum similarity against the seed that must be satisfied. When creating models simultaneously in multiple channels, the joint similarity, or the average of the similarities for each channel, is used. The resultant models and the registered particles that contributed to them can then be further analyzed.

## Protocol

NOTE: This protocol and video supplement the original paper^10^ describing the software package in more detail. Readers are encouraged to review this carefully for additional guidance regarding the use of the software. There are three main stages: particle extraction, which segments large images into individual particles; seed selection, where common structures are identified in the data and aligned to produce seeds, which are used in the final stage; and model generation, where template matching based on these seeds aligns the extracted particles and averages a subset to produce the SPA models.

### 1. Setup Prior to Running the Software Package

1. Prepare samples of the structure under study on a coverslip or in the relevant experimental conditions.
2. Image the samples with super-resolution fluorescence microscopy, such as structured illumination microscopy (SIM)^11^ or stimulated emission depletion (STED)^12^ microscopy. NOTE: The precise details of how to prepare and image samples depends greatly on the nature of the structure under study, so the relevant literature should be consulted. As an example, the precise method for preparing and imaging samples of vaccinia virus, such as those used here, is described in the representative results section.
3. Create images of multiple fields of view showing a large number of separate copies of the structure or particles, preferably thousands. Image particles that are as well separated from each other as possible and ensure that the images are free of dirt or other fluorescent structures that are not of interest.
4. Open all images containing the particles in Fiji by dragging the files into the Fiji toolbar or by selecting "File">"Open".
5. Select "Image">"Stack">"Tools">"Concatenate" to concatenate the images into a single stack. Then, if the resultant image is a Hyperstack, turn it into a stack by selecting "Image">"Hyperstacks"> "Hyperstack to Stack". ​NOTE: The final stack should have intercalated channels. If there are two channels, the first slice in the stack should be channel 1 from the first image, the second slice should be the corresponding channel 2, the third slice should be channel 1 from the second image, and so on.

### 2. Extract the Particles

1. Choose the image to segment and select "Extract Viral Structures". Choose where to save the extracted particles and view the "Extract Viral Structures"**dialog (**Figure 2**).
2. Assign extraction parameters with initial estimates by entering them into the "Extract Viral Structures" dialog as follows. Fine-tune these parameters after previewing the image segmentation.
  1. Set the number of channels in the dataset as the number of different fluorescence channels that have been imaged (*e.g.,* 2).
  2. Set the reference channel from which particles are extracted by detection of peaks in that channel by entering the number of the choice of channel. Select the most consistent channel; that is, the channel in which the most particles have the same appearance. ​NOTE: If possible, particles in this channel will have a central maximum.
  3. Choose whether or not to apply a pre-detection Gaussian blur. Set the pre-detection Gaussian blur to 0 to apply no blur; if this value is increased, a Gaussian blur filter of the given radius is applied before local maxima detection. Use this feature if the reference channel does not have a central maximum (*e.g.,* ring shape); blurring induces the appearance of one. ​NOTE: Segmented regions of interest (ROIs) do not have the Gaussian blur applied to them, as this feature is only used to position ROIs in the reference channel.
  4. Set the ROI radius (which will be set around each local maximum) in pixels. Choose a value such that that ROIs are slightly larger than the largest particles, such as in **Figure 3**. For example, if the largest particles appear to have a diameter of around 30 pixels (estimated roughly by eye), then set the ROI radius to 20 pixels.
  5. Set the number of ROIs to use per frame to an initial, relatively small value below 100.
  6. Set the maximum ROI overlap. If the particles are well separated, keep this small; if particles are clustered, increase this to enable the ROIs to overlap.
3. Select "show preview". NOTE: When this is selected, the extracted ROIs for the reference channel associated with the current frame will appear.
4. Adjust the ROI radius, the number of ROIs, and the maximum ROI overlap to have ROIs of suitable size around as many particles as possible, as in **Figure 3**.
5. Select "OK" to run the segmentation. Close the image and the ROI manager. ​NOTE: Do not change the names of the files of the particle sets. These names must be in the format "Viral particles - channelX" for the following sections.

### 3. Select Seeds

1. Select "Generate Seeds", select the folder where the extracted particles are saved, and view the "Generate Seeds" dialog and windows (**Figure 4**).
2. Assign initial seed-selection parameters by entering them into the "Generate Seeds" dialog, as follows.
  1. Set the reference channel that will be fitted to align and center all channels. Choose a channel in which most particles have the same appearance and that has a central maximum, if possible.
  2. Select the boxes for all channels for which a seed should be generated.
  3. Select if the seeds should be rotated by 90°. Use this feature to have the alignment consistent with other models
  4. Choose whether or not to apply a pre-alignment Gaussian blur. Set the blur radius for each channel to 0 to apply no blur. Increase this value to apply a Gaussian blur filter of the given radius before realignment. Use this feature if the particles do not have a central maximum; blurring induces the appearance of one. ​NOTE: Seeds will not have the blur applied, as this feature is only to get consistent alignment.
  5. Select whether to use shift correction to separately center seeds for non-reference channels, although not rotate them, by checking or unchecking the "Shift correction" boxes for each channel. NOTE: Use this if the channels are not well aligned with each other; it can also be useful for aligning other channels solely according to the reference, without shift correction.
3. Choose particles to use as seeds. Search through the particle sequence to find a particle that resembles the structure that is to be modeled and record the frame number.
4. Enter the number in the "Frames to use" box, and more windows will appear (**Figure 5**). Enter multiple frame numbers separated by commas.
5. View the seed frames and the resultant average seeds in the windows that appear. NOTE: The log will suggest seeds similar to the average.
6. Adjust the reference channel, the Gaussian blur radius, and the shift correction options to optimize the seed selection process so that a number of seed frames found have a similar appearance. Continue adding seeds until average seeds are created that satisfactorily resemble the structure seen in the data that is to be modeled.
7. Name the seeds and where they will be saved to and select "OK" to save the final seed images for later use in model generation.

### 4. Generate Models

1. Select "Generate Models Based On Seeds", select the folder where the extracted particles are saved, and view the "Generate Models" dialog (**Figure 6**).
2. Load the seeds for each channel by using the checkboxes. ​NOTE: Seeds will be saved in a subfolder that was named in the "Generate Seeds" dialog. The default name is "Analysis". Take care to select the seed *Averages*, not the Frames, which are also saved for reference.
3. Assign initial model generation parameters by entering them into the "Generate Models" dialog, as follows. Fine-tune these parameters later.
  1. Select whether to use a reference channel for alignment, which calculates translations and rotations only from the reference channel and applies them to all channels. If using this option, select the channel to use as the reference. ​NOTE: This option should only be chosen to specifically use one channel as a reference, such as if doing reference-based structure discovery. Otherwise, it will result in less accurate models. The channels should be well aligned if using this option.
  2. Select whether to square image intensities during template matching. This will accentuate small differences, so use this when creating a model which has particularly subtle features.
  3. Choose a minimum similarity against seed by using the "Minimum similarity against seed" slider or by entering a number into the box. ​NOTE: Only particles with a similarity to the seeds greater than this cut-off will be used. 60-80% is typically a good choice to start with.
  4. Set the maximum number of iterations to 1, optimize the minimum similarity against seed, and increase it later, as required.
  5. Select the boxes to choose the elements of the model generation process that will appear. NOTE: "Show seeds" will display the seeds that have been loaded with the boxes at the top of the dialog. "Show models" will display all iterations of the models that are created from averaging the subset of particles that meets the minimum similarity against seed. "Show MSE" will display a mean squared error (MSE) image that highlights the areas of the model that are most variable. "Show particles" will display the subset of particles that are used to create the models, registered according to the highest iteration of the model that is displayed.
4. Select "Show preview" to generate preview models and view the results. NOTE: This is the most computationally intensive step of the process. The running time for a set of a few thousand particles with diameters of a few tens of pixels on a desktop PC should be around 10 min. If computation time is an issue, users should first try the algorithm on a smaller subset of the data or use a smaller ROI radius in step 2.2.4, if possible.
5. View the generated models and optimize the parameters, especially the minimum similarity against seed. Increase the minimum similarity against seed until only true particles of the modeled morphology are included in the model.
6. Increase the maximum number of iterations by using the "Maximum number of iterations" slider or by entering a number into the box and allow the model generation process to iterate. Use a value around 10 for maximal iteration.
7. Name models and select "OK" to save model evolution stacks that contain all iterations of the final model generation process. NOTE: If the minimum similarity against seed is so high that no particles have this similarity, nothing will be updated. If the plugin appears to be frozen, consider the possibility that the minimum similarity is too high.

## Representative Results

Here, we demonstrate the software on the model poxvirus, vaccinia virus. One of the most complex mammalian viruses, vaccinia packages around 80 different proteins within a 350 x 270 x 250 nm^3^ brick-shaped particle^13^,^14^. Three substructures are discernible by electron microscopy: a central core, which contains the dsDNA genome; two proteinaceous structures, called lateral bodies, which flank the core; and a single proteolipid bilayer envelope^15^. The large size, complex structure, and amenability to recombinant fluorescent protein tagging make vaccinia an excellent system to demonstrate the VirusMapper workflow.

Using the software as described here, the distribution of a variety of proteins on the vaccinia virion can be modeled. A protein was labeled and imaged, possibly in combination with another protein of known distribution as a reference, and the software was used as described to produce average models of the localization of that protein on the particle. In this example, two proteins were modeled, the inner core protein L4, and the major lateral body component F17.

A recombinant vaccinia virus which has F17 tagged with GFP and L4 tagged with mCherry^16^ was used. Purified virus was diluted in 1 mM Tris, pH 9, and bound to washed, high-performance coverslips by coating them for 30 min at room temperature. The samples were then fixed by applying 4% formaldehyde in PBS for 20 min. Coverslips were mounted immediately onto slides in antifade mounting medium. Imaging was carried out by SIM on a commercial SIM microscope. A field of view was selected containing hundreds of viruses and images were acquired using 5 phase shifts and 3 grid rotations with 561 nm (32 µm grating period) and 488 nm (32 µm grating period) lasers. Images were acquired using a sCMOS camera and processed using the microscope software. Channels were aligned based on a multi-colored bead slide imaged with the same image acquisition settings. After SIM reconstruction and channel alignment images were opened in Fiji and concatenated into a single image stack.

Viral particles were extracted from the images using the L4 channel as the reference and without applying any Gaussian blur, as these particles have a central maximum. Around 15,000 particles were extracted in this experiment.

Due to the geometry of vaccinia, the lateral bodies have a distinctly different appearance based on the virus orientation. We visualized two orientations in which either one or two lateral bodies could be distinguished. We referred to these orientations as frontal and sagittal, respectively.

Separate seeds for the frontal and sagittal orientations were selected by searching through the particle list at the "Generate Seeds" stage (**Figures 4** and **5**); particles that were clearly in one orientation or the other were chosen. The L4 channel was used as the reference channel to align the seeds with one another. Again, no Gaussian blur was necessary. 5 particles for each orientation were selected and were averaged to produce the seeds.

Models were generated for each orientation based on these seeds. Neither a reference channel nor squared intensity values were used. The maximum number of iterations was set initially to 1, and the minimum similarity was set to include around 1,000 particles in each case, which gave a consistent appearance for each orientation. The maximum number of iterations was then increased to allow for the convergence of the model. Models were thus generated for the two orientations in the two channels (**Figure 7)**.


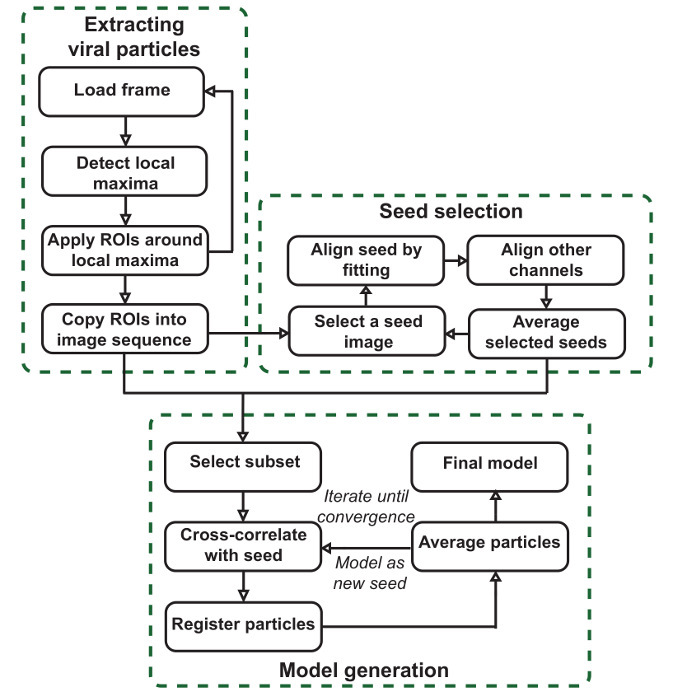
**Figure 1:VirusMapper workflow.** The plugin is organized into three main stages. Viral particles are extracted from large images, template images or seeds are selected semi-manually from the data, and final SPA models are generated from the data by referring to the seeds. Please click here to view a larger version of this figure.


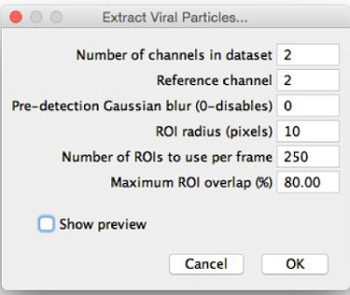
**Figure 2:"Extract Viral Structures" dialog. **When selecting "Extract Viral Structures", this dialog will appear. The parameters should be filled with initial estimates for optimal segmentation. "Show preview" can then be selected, allowing the ROIs to be previewed and the parameters to be fine-tuned. Please click here to view a larger version of this figure.


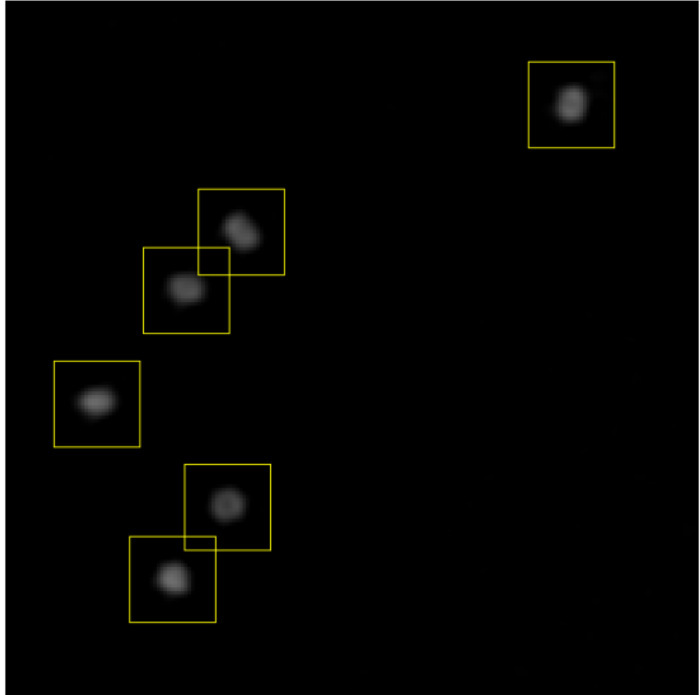
**Figure 3:Setting extraction parameters. **After previewing the ROIs that will be extracted, the ROI radius, number of ROIs, and maximum ROI overlap are adjusted to achieve a situation like this. ROIs are slightly larger than the particles, all particles are included in an ROI, and ROIs can overlap sufficiently to allow clustered particles to be separated. Please click here to view a larger version of this figure.


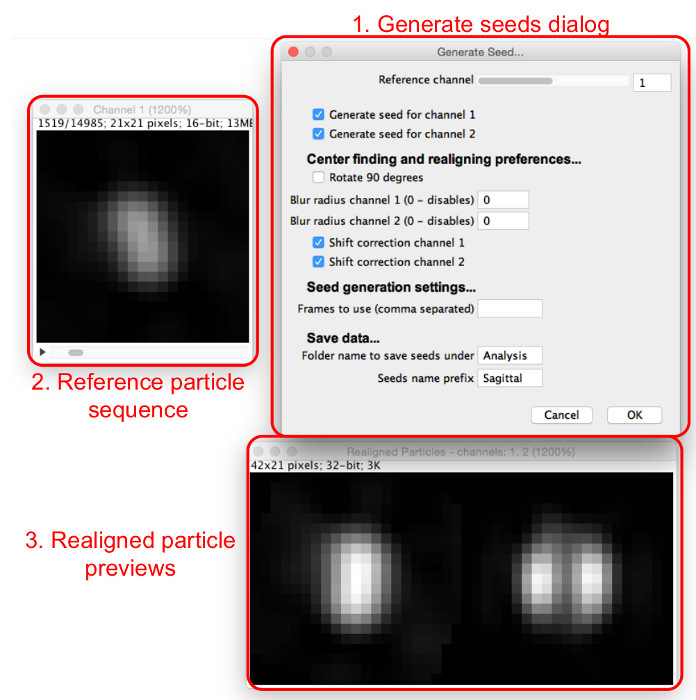
**Figure 4:Generating template matching seeds.** The "Generate Seeds" dialog (1) sets out the parameters to be assigned. The reference particles sequence (2) allows the user to scan through particles in the reference channel. When a particle is viewed in the reference particles sequence, realigned particles for all channels can be viewed in the realigned particle previews (3). Please click here to view a larger version of this figure.


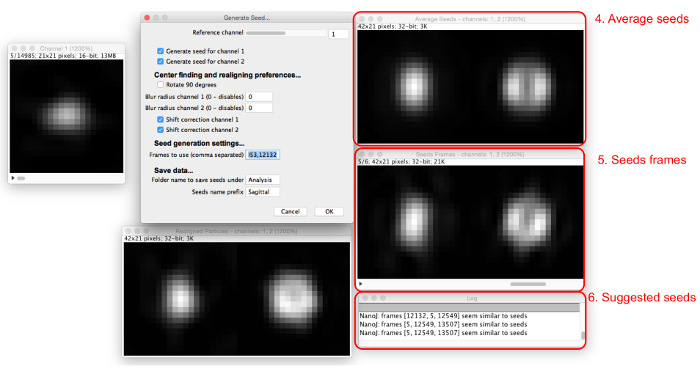
**Figure 5:Adding seed images.** As seeds are added to the "Frames to use" box, the average of all seeds (4) and the frames involved (5) are displayed. Particles which are similar to the current average seeds are suggested in the dialog box (6). Please click here to view a larger version of this figure.


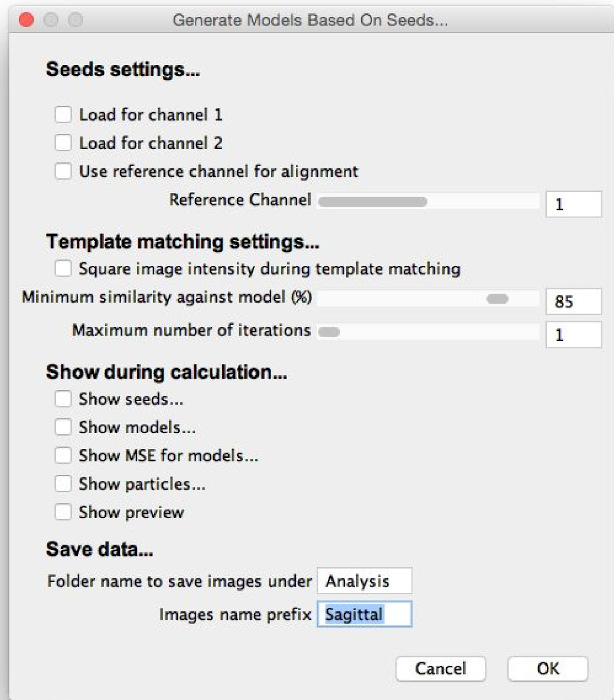
**Figure 6:"Generate Models" dialog. **When selecting "Generate Models Based on Seeds," this dialog will appear. The parameters should be filled with initial estimates for optimal model generation, and the elements of the model generation procedure to be shown during calculation should be selected. "Show preview" can then be selected, allowing the model generation process to run and the parameters to be fine-tuned. Please click here to view a larger version of this figure.


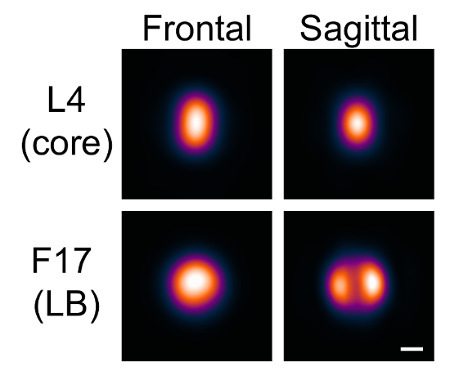
**Figure 7:Models generated with VirusMapper. **Vaccinia virions with the L4 core protein tagged with mCherry and the F17 lateral body protein tagged with EGFP were imaged using SIM. Models were then generated with the software, as described in the protocol. Two orientations, frontal and sagittal, are distinguished by the appearance of the lateral bodies. Scale bar = 100 nm. Please click here to view a larger version of this figure.

## Discussion

With this method, researchers are equipped to combine the power of SPA and SR microscopy in order to generate high-precision, multi-channel 2D models of the protein architecture of viruses and other macromolecular complexes. However, some important considerations should be taken into account.

Seeds should be chosen to represent a structure that is consistently seen. Thus, the raw data should be inspected carefully before the seeds are chosen. This is important for preventing biased models. Choices can be validated by the examination of the minimum similarity thresholds needed to include a certain number of particles in the models. Clearly, for a choice of seed, the higher this threshold needs to be for a given number of particles, the more that structure is apparent in the data.

The template matching concept is particularly useful when there is heterogeneity in the data. All different structures that are visible should be identified and different models created for each case. By separating heterogeneous structures in one channel but simultaneously creating models in a second channel, patterns may emerge that would not have been immediately evident.

Another consideration to be aware of when using this algorithm is that the iteration procedure will maximize stochastic asymmetry. For example, when modeling a structure with two symmetric maxima, all slight asymmetries between the maxima will be aligned with each other during the iteration, and the final model will thus be maximally asymmetric. If this does not reflect a known symmetry in the structure being modeled, then this should be taken into account. Currently, the only way to avoid this maximization is to limit the number of iterations to 1, although a potential development would be for VirusMapper to incorporate axes of symmetry into the model generation process. Any new versions of VirusMapper will be available on the referenced website (see **Materials Table**). Users will also find a FAQ here to answer any common queries.

The software as described is applicable to any structure that can be imaged with sufficient resolution to visualize the features that the user wishes to model. Although SPA can improve resolution, it clearly will not improve the visibility of features that are otherwise not visible. This protocol is not, therefore, a method to improve the quality of data. As with any technique, careful sample preparation and optimization of imaging strategy will provide the cleanest data and the best resultant models.

The choice of SR imaging modality is also important and, in general, will depend on the sample at hand. VirusMapper has been validated to work well with SIM and STED^10^, and it can also be used with high-quality localization microscopy data, but care should be taken in this case, as sparse labeling could cause issues similar to those of asymmetry maximization.

Currently, VirusMapper is the only freely available algorithm for the single-particle analysis of fluorescence images and the only general-purpose 2D SPA averaging software. Other studies that have made use of the same principles^4^,^6^,^8^ have used custom software specialized for each particular study. General-purpose algorithms for the reconstruction of 3D data have been published^5^,^18^, although no software was provided.

When used as described in this article, VirusMapper can be used to produce precise, accurate, and robust models of the macromolecular protein architecture of viruses and other complexes. With these models, researchers can take precise measurements of the average dimensions of the structures under study, potentially allowing them to reach biological conclusions that would not have otherwise been possible.

Furthermore, with the multi-channel capabilities of this technique, it is possible to map an unlimited number of proteins and components within complexes and to discover novel protein organization. Examining changes in nanoscale structure in different biologically relevant conditions, such as different stages of a virus life cycle, has the potential to offer valuable insights into biology.

## Disclosures

The authors have nothing to disclose. 
